# Emergency Department Patient Satisfaction Scores Are Lower for Patients Who Arrive During the Night Shift

**DOI:** 10.5811/westjem.20326

**Published:** 2024-10-02

**Authors:** Tony Zitek, Luke Weber, Tatiana Nuñez, Luis Puron, Adam Roitman, Claudia Corbea, Dana Sherman, Michael Shalaby, Frayda Kresch, David A. Farcy

**Affiliations:** * Mount Sinai Medical Center, Department of Emergency Medicine, Miami Beach, Florida; † Florida International University, Herbert Wertheim College of Medicine, Miami, Florida

## Abstract

**Background:**

Increasingly, patient satisfaction scores are being used to assess emergency physicians. We sought to determine whether the patient satisfaction scores collected by our hospital system are lower for patients who are treated in the emergency department (ED) on night shifts as compared to those treated on day shifts.

**Methods:**

We performed a cross-sectional analysis of patient satisfaction scores from three EDs in Florida. We obtained satisfaction data from NRC Health (the company that provides our surveys) using a random sample of 1,000 completed surveys from patients treated in 2022; we also performed manual chart review to obtain clinical data. The satisfaction surveys asked patients how likely they would be to recommend the facility (from 0–10). Patients who provided a score of 9 or 10 were considered “promoters.” For our primary analysis, we compared the percentage of promoters for the day shift encounters (7 am to 7 pm) to the night shift encounters (7 pm to 7 am). We also performed a multivariable logistic regression analysis using several demographic and clinical variables to further assess the association between night shift arrival and satisfaction scores.

**Results:**

Of the 1,000 surveys analyzed, 66.3% of patients arrived during the day shift, and 33.7% arrived during the night shift. Of those who arrived during the day shift, 525 (79.2%) were promoters compared to 228 (67.7%) of those who arrived during the night shift, a difference of 11.5% (95% confidence interval [CI] 5.7–17.4%), *P* < 0.001. On multivariable analysis, night shift arrival was associated with a lower chance of a patient being a promoter, with adjusted odds ratio 0.60 (95% CI 0.43–0.84), *P* = 0.003.

**Conclusion:**

Patients who presented to the ED during the night shift were less likely to be promoters than patients who arrived during the day shift. Assessments of patient satisfaction data should account for time of visit and other facility-related and operational characteristics.

Population Health Research Capsule What do we already know about this issue?

*Physicians are judged based on patient satisfaction scores. Prior data found that certain patient and facility characteristics are associated with satisfaction scores.*

What was the research question?

*Do patients who present to the ED at night provide lower satisfaction scores than patients who present during the daytime?*

What was the major finding of the study?

*Of day shift patients, 79.2% were “promoters” vs 67.7% of night shift patients (difference 11.5% [95% CI 5.7–17.4%]),* *P < 0.001.*

How does this improve population health?

*This data helps us better interpret patient satisfaction data, which may help improve our ability to provide patient-centered care.*

## INTRODUCTION

With emergency departments (ED) open 24/7, most emergency physicians work some night shifts. Unfortunately, prior data has shown that night shift work is associated with increased risk of a variety of medical conditions^1^ ^–^ ^5^ and motor vehicle collisions after those shifts.^6^ Additionally, emergency physicians working at night may have to deal with reduced support staff, tired patients, and fewer available consultants. Moreover, multiple prior studies have demonstrated that while on night shift, cognitive performance declines.^7^ ^,^ ^8^

Despite the unique challenges of night shifts, emergency physicians are generally held to the same standards on night shifts as they are on day shifts, and one way they are now assessed is by patient satisfaction scores. Indeed, patient satisfaction has become an increasingly important part of healthcare in large part because of the incentives initiated by the Affordable Care Act in 2010^9^; now, both institution and physician payment are sometimes based on patient satisfaction scores.^10^

Prior studies have shown that certain factors including shorter ED length of stay (LOS),^11^ ^–^ ^13^ older patient age,^14^ and good communication^15^ are associated with better ED patient satisfaction scores. Two prior studies have investigated the relationship between treatment during night shifts and patient satisfaction scores.^14^ ^,^ ^16^ One found no statistically significant association,^14^ while another found that physicians who worked fewer night shifts had higher patient satisfaction scores.^16^ Given the conflicting evidence to date and the increasing emphasis on patient satisfaction, we felt that additional study was warranted to assess the relationship between night shift work in the ED and patient satisfaction.

Our primary objective in this study was to determine whether patients who are cared for during night shifts provide lower patient satisfaction scores than those cared for during day shifts, using the real-world satisfaction data. Secondarily, we sought to determine whether other demographic and clinical characteristics are associated with ED patient satisfaction scores.

## METHODS

### Study Design and Setting

We performed a cross-sectional analysis of ED patient satisfaction scores from patients who presented to a single hospital system in the State of Florida in the southeastern United States from January 1–December 31, 2022. Specifically, we performed a secondary analysis of a previously collected dataset of satisfaction scores, and we performed a chart review to supplement that data. We followed the Strengthening the Reporting of Observational Studies in Epidemiology (STROBE) guidelines. The study was approved by the Mount Sinai Medical Center Institutional Review Board. This study received no external funding.

Our hospital system has a tertiary care, community teaching hospital located in Miami Beach, Florida, as well as a freestanding ED located in Hialeah, Florida, (freestanding ED #1) and a freestanding ED located in Aventura, Florida, (freestanding ED #2). The main hospital’s ED had 56,005 visits during 2022, while freestanding ED #1 had 37,932 visits and freestanding ED #2 had 19,635 visits. Emergency medicine residents work shifts only at the main hospital’s ED. Advanced practice practitioners (APP) work shifts at all three facilities. Shift times are shown in Table 1. In 2022, three emergency physicians only worked only night shifts, and one physician worked only day shifts. Some attending physicians and APPs only worked at one facility; others worked at two or all three.

**Table 1.** Emergency department staff shift times in 2022.

	Main ED	Freestanding ED #1	Freestanding ED #2
Nursing shifts	7 am to 7 pm 10 am to 10 pm 2 pm to 2 am	7 am to 7 pm 10 am to 10 pm 2 pm to 2 am	7 am to 7 pm 10 am to 10 pm 2 pm to 2 am
Attending physician shifts	7 am to 3 pm 10 am to 10 pm 11 am to 9 pm 2 pm to midnight 9 pm to 7 am	7 am to 7 pm 11 am to 11 pm 7 pm to 7 am	7 am to 7 pm 7 pm to 7 am
Resident shifts	All shifts except 7 am to 3 pm on Wednesdays^*^	None	None
Advanced practice practitioner shifts	10 am to 10 pm	9 am to 9 pm 2 pm to 2 am	10 am to 10 pm

* Residents are not in the ED on Wednesday mornings from 7 am to 1 pm due to academic conference.

*ED*, emergency department.

### Selection of Participants

In 2022, NRC Health (Lincoln, NE) administered our patient satisfaction surveys and tracked satisfaction data. Surveys were sent by both text message and email to all patients who left the ED. All patients who completed the NRC Health ED patient satisfaction survey in 2022 were eligible for inclusion in this study. Admitted patients were not sent surveys and were excluded from analysis.

### Measurements

NRC Health keeps a database with the responses from satisfaction surveys and demographic information about the patients who complete the surveys. In our hospital system, currently, individual physician-level patient satisfaction scores are tracked and assessed using these data, but compensation is not dependent upon them. We generated a report from NRC Health’s data for all patients who completed a satisfaction survey during 2022 and then used a random number generator to create a sample of 1,000 patient encounters for analysis. For each of these patient encounters, two medical students transferred patient responses and available demographic information into a spreadsheet in Microsoft Excel v16.79.1 (Microsoft Corp, Redmond, WA). In particular, NRC Health provided us with the following data for each encounter: the date of the visit; the facility; the name of the physician or APP; method of patient response (email or text message); and the patient’s age, sex, race, medical record number, address, marital status, and preferred language. The two medical students who abstracted these data points had no role in the abstraction of the other data discussed below.

Next, we created a separate spreadsheet with additional clinical information for each of the 1.000 patient encounters using our electronic health record system (EHR) (Epic Systems Corporation, Madison, WI). Six abstractors (three emergency medicine residents, two emergency attendings, and one nurse practitioner) performed manual chart review to determine the patient’s ethnicity, mode of arrival to the ED, times of arrival and departure, Emergency Severity Index (ESI) score,^17^ disposition, clinician who discharged the patient (and their supervising attending, if applicable), and whether or not each of the following was performed during the patient encounter: resident participation; sign-out; blood test; advanced imaging; in-person consultant evaluation, consultation by phone (only); opioid pain medicine administration; and prescription provided.

In general, manual chart review followed the methods suggested by Kaji et al.^18^ The abstractors who performed manual chart review were blinded from the satisfaction data. None of the abstractors or investigators have been a nocturnist. The abstractors filled in 15 columns in the spreadsheet with the data points above. They were trained on proper data abstraction by the principal investigator (TZ), and they followed a data dictionary that explicitly defined the variables and explained where to find them in the EHR. The data dictionary is included as an appendix, which provides detailed definitions of all variables. The definitions of a few important variables are also defined here as follows:

We considered patients to have arrived during the day shift if they arrived in the ED between 7 am–7 pm and to the night shift if they arrived between 7 pm–7 am. We chose these definitions because many physician and nursing shifts follow these time schedules in our system (Table 1). We also divided patients into the time of year they came to the ED by standard quarters.

The type of clinician (physician or APP) who evaluated the patient primarily was determined based on the name of the clinician on the survey as per NRC Health data. For example, a patient was considered to have been seen primarily by an APP if the APP was the person listed on the satisfaction survey. As mentioned above, we also manually recorded the name of the clinician (and their supervising attending) who discharged the patient for each patient encounter. For patients who were not signed out, the discharging clinician (or supervising attending) was fully consistent with the listed name on the surveys. However, for patients who were signed out, sometimes the initial clinician who treated the patient was listed on the survey and sometimes a subsequent one was. Since administrators assess the satisfaction data based on the name of the clinician on the surveys, we used the name of the clinician on the survey as the primary treating clinician.

Patients who left the ED before being evaluated by a physician or APP could still be included in the study if they completed a satisfaction survey but were considered to have not been seen by a physician or APP. All six abstractors obtained the data for a group of the same 50 patients to allow for an assessment of the inter-rater reliability. We calculated the free-marginal kappa for the two variables that we considered to be the most difficult to abstract: sign-out, and in-person evaluation by consultant.

After completing data collection, the principal investigator (TZ) merged the spreadsheets with the satisfaction data and the clinical data, and the data was analyzed as described below.

### Outcomes

In 2022, our administration considered the most important question on the satisfaction surveys to be: “How likely is it that you would recommend [facility name] to a friend or colleague?” (from 0–10). A patient who provided a score of 9 or 10 was considered to be a “promoter”; a score of 7 or 8 was considered “passive”; and a score of 0–6 was considered to be a “detractor.” The percentage of promoters minus the percentage of detractors is deemed the “net promoter score,” which is used to measure overall satisfaction in healthcare as well as in other businesses.^19^ ^,^ ^20^ Our primary outcome was the percentage of completed patient satisfaction surveys that qualified as promoters. Secondarily, we determined the net promoter score and the adjusted odds ratios for being a promoter for several demographic and clinical variables.

### Analysis

Based on a preliminary analysis of NRC Health data, we anticipated that there would be approximately twice as many completed surveys from patients who arrived during the day shift vs the night. Additionally, we knew that approximately 75% of our completed surveys in 2022 qualified as promoters. Based on gestalt, we hypothesized that the percentage of promoters from the day shift would be eight points higher than the night shift. To test our hypothesis with an alpha of 0.05 and power 0.8, we required responses from 957 patients. We rounded this up to 1,000 and chose that as our sample size.

For our primary analysis, we compared the percentage of promoters for patient encounters in which the patient arrived during the day shift compared to night shift. We made the unadjusted comparison our primary analysis since that is how patient satisfaction scores are being used to assess emergency physicians in our hospital system. We also compared the demographic and clinical characteristics of the patient encounters for the two groups. We determined normality with the Shapiro-Wilk test. For normal distributions, we compared the means of groups using *t*-tests. For non-normal distributions, we compared medians using the median test. We used the Fisher exact test to compare categorical variables.

Secondarily, given that a patient who arrives during the end of a day shift might be mostly treated by the night staff (or vice versa), we also analyzed patients based on the time of ED departure. In other words, we considered night shift patients to be those who departed between 7 pm – 7 am. Lastly, to further isolate the night-time hours when people are generally sleeping, we divided patients by arrival time into three eight-hour epochs: 6 am – 2 pm (day), 2 pm – 10 pm (swing), and 10 pm – 6 am (night).

Lastly, we performed a multivariable logistic regression analysis with “promoter” (yes or no) as the dependent variable. Based on prior data,^11^ ^–^ ^14^ ^,^ ^16^ ^,^ ^21^ ^–^ ^23^ we included the following variables in our model: ED LOS (continuous); ED site (categorical); elderly (age > 65) (binary); pediatric (binary); race (White or not); ethnicity (Hispanic or non-Hispanic), health insurance type (no insurance, commercial, or government/other); non-English speaking (binary); Emergency Severity Index (ESI) (2 or 3 vs 4 or 5); and advanced imaging performed (binary). Based on investigator hypothesis, we also chose to include the following as covariates: quarter of the year (1, 2, 3, or 4); married (binary); seen by a resident (binary); seen primarily by an APP (binary); arrival by ambulance (binary); and blood test performed (binary). We also hypothesized that consultant evaluations would be associated with better satisfaction scores, but these occurred too rarely in our dataset to be included in the regression analysis. We used the Hosmer-Lemeshow statistic to assess goodness of fit of the regression model.

Data was aggregated in Excel and analyzed in R Studio v2023.03.0 (RStudio PBC, Boston, MA). Using two-sided hypothesis tests, we considered *P* < 0.05 to be statistically significant.

### Missing Data

In a few cases, race and ethnicity were not recorded. This was handled as follows: Patients who were documented as having White or Caucasian race were considered to be “White.” Patients who were documented as Black or African American were considered to be “Black.” Patients documented as Asian, American Indian, multiracial, other, or for whom race was not documented were considered neither White nor Black. Similarly, if a patient was documented as “Hispanic,” their ethnicity was considered to be “Hispanic.” If they were documented to be non-Hispanic or if their ethnicity was not documented, they were considered “non-Hispanic.”

## RESULTS

### Overall

As shown in the Figure, 6,375 satisfaction surveys were completed in 2022, and we randomly selected 1,000 for analysis. Of these, 824 patients responded by text message and 176 responded by email. Our data included surveys evaluating 44 different attending physicians and 18 APPs. There were no missing data points, except for five patients for whom no race and ethnicity were recorded. Inter-rater reliability for the two assessed variables was almost perfect with free-marginal kappa 0.98 (95% confidence interval [CI] 0.94–1.0) for sign-out and 0.98 (95% CI 0.9–1.0) for in-person evaluation by a consultant. Overall, 75.3% of patient encounters qualified as promoters, and net promoter score was 57.8.

**Figure.**
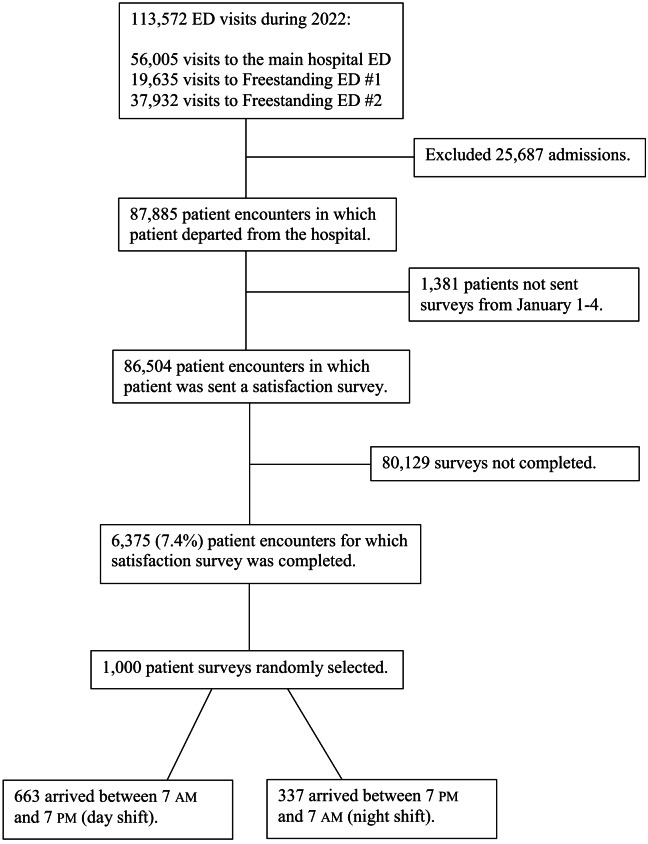
Flow of patient encounters.

### Night vs Day Shifts

In total, 663 (66.3%) patients arrived during the day shift, and 337 (33.7%) arrived during the night shift. Table 2 shows a comparison of characteristics of these two groups. Notably, the groups were not balanced on several characteristics including age, race, marital status, insurance, type of clinicians involved, ESI, and advanced imaging performed. Regarding the primary outcome, 525 (79.2%) of those who arrived during the day shift were promoters compared to 228 (67.7%) of those who arrived during the night shift, an absolute difference between groups of 11.5% (95% CI 5.7–17.4%), *P*  < 0.001. The net promoter score for the day shift was 64.9 and 44.0 for the night shift. Data stratified by facility are shown in Table 3.

**Table 2.** Characteristics of patient visits for patients who arrived during the day shift (7 am to 7 pm) as compared to those who arrived during the night shift (7 pm to 7 am).

	Day-shift arrival (n = 663)	Night-shift arrival (n = 337)	Absolute difference (95% CI)	*P*-value
Patient demographics				
Mean age (SD)	49.3 (22.2)	43.0 (23.4)	6.3 (3.2 to 9.3)	<0.001^*^
Pediatric (< 18 years), n (%)	60 (9.0)	46 (13.7)	4.7 (0.3 to 8.9)	0.03^*^
Elderly (> 65 years), n (%)	203 (30.6)	78 (23.2)	7.4 (1.8 to 13.2)	0.01^*^
Male, n (%)	273 (41.2)	147 (43.6)	2.4 (−4.0 to 8.9)	0.46
Race White, n (%)	530 (79.9)	248 (73.6)	6.3 (0.7 to 12.0)	0.02
Race Black, n (%)	53 (8.0)	31 (9.2)	1.2 (−2.5 to 4.9)	0.52
Hispanic, n (%)	365 (55.1)	205 (60.8)	5.8 (−0.7 to 12.2)	0.08
Commercial health insurance, n (%)	391 (59.0)	203 (60.2)	1.2 (−5.2 to 7.7)	0.70
No health insurance, n (%)	53 (8.0)	48 (14.2)	6.3 (2.0 to 10.5)	0.002^*^
Married, n (%)	267 (40.8)	99 (29.6)	11.1 (5.0 to 17.3)	<0.001^*^
Non-English speaking, n (%)	228 (34.4)	114 (33.8)	0.6 (−5.7 to 6.8)	0.86
From out of state, n (%)	41 (6.2)	23 (6.8)	0.6 (−2.6 to 3.9)	0.70
Time of year of visit				
Quarter 1, n (%)	155 (23.4)	82 (24.3)	1.0 (−4.7 to 6.6)	0.74
Quarter 2, n (%)	186 (28.1)	87 (25.8)	2.2 (−3.6 to 8.0)	0.75
Quarter 3, n (%)	157 (23.7)	83 (4.6)	1.0 (−4.7 to 6.6)	0.74
Quarter 4, n (%)	165 (24.9)	85 (25.2)	0.3 (−5.4 to 6.0)	0.91
Clinician and facility characteristics				
Main hospital	388 (58.5)	204 (60.5)	2.0 (−4.4 to 8.4)	0.54
Freestanding ED #1	160 (24.1)	67 (19.9)	4.3 (−1.1 to 9.6)	0.13
Freestanding ED #2	115 (17.4)	66 (19.6)	2.2 (−2.9 to 7.4)	0.38
Resident participated, n (%)	217 (32.7)	179 (53.1)	20.4 (14.0 to 26.8)	<0.001^*^
Advanced practice practitioner, n (%)	171 (25.8)	36 (10.7)	15.1 (10.4 to 19.8)	<0.001^*^
Sign-out, n (%)	24 (3.6)	30 (8.9)	5.3 (1.9 to 8.6)	<0.001^*^
Clinical characteristics				
ESI^a^ 2, n (%)	20 (3.0)	13 (3.9)	0.9 (−1.6 to 3.3)	0.48
ESI 3, n (%)	401 (60.5)	229 (68.0)	7.5 (1.3 to 13.7)	0.02^*^
ESI 4, n (%)	239 (36.1)	93 (27.6)	8.5 (2.4 to 14.5)	0.007^*^
ESI 5, n (%)	3 (0.5)	2 (0.6)	0.1 (−0.8 to 1.1)	0.77
Arrived by ambulance, n (%)	24 (3.6)	22 (6.5)	2.9 (0.0 to 5.9)	0.04^*^
Blood test performed, n (%)	287 (43.3)	126 (37.4)	5.9 (−0.5 to 12.3)	0.07
Advanced imaging performed, n (%)	228 (34.4)	90 (26.7)	7.7 (1.7 to 13.6)	0.01^*^
In-person consultant evaluation, n (%)	20 (3.0)	8 (2.4)	0.7 (−1.4 to 2.7)	0.56
Phone (only) consultation, n (%)	23 (3.5)	2 (0.6)	2.9 (1.3 to 4.5)	0.006^*^
Opioid pain medicine given, n (%)	72 (10.9)	36 (10.7)	0.2 (−3.9 to 4.2)	0.93
Given prescription, n (%)	374 (56.4)	170 (50.4)	6.0 (−0.6 to 12.5)	0.07
Median length of stay (IQR), min	194 (123–259)	184 (123–265)	10 (−7 to 28)	0.28
AMA, eloped, or LBT^b^, n (%)	18 (2.7)	17 (5.0)	2.3 (−0.3 to 5.0)	0.06

a Emergency Severity Index. There were no patients with an ESI of 1.

b All other patients were discharged except for two who were transferred to other hospitals.

* Indicates a statistically significant difference between groups.

*AMA*, against medical advice; *CI*, confidence interval; *ED*, emergency department; *ESI*, Emergency Severity Index; *IQR*, interquartile range; *LBT*, left before treatment.

**Table 3.** The percentage of completed satisfaction surveys considered promoters overall and at each of the three emergency departments, comparing patients who arrived on day shift vs night shift.

	Day-shift arrival promoters, n (%)	Night-shift arrival promoters, n (%)	Absolute % difference (95% CI)	*P*-value
Overall (N = 1,000)	525 (79.2)	228 (67.7)	11.5 (5.7 to 17.4)	<0.001^*^
Main hospital (n = 592)	304 (78.4)	137 (67.2)	11.2 (3.6 to 18.8)^*^	0.003^*^
Freestanding ED #1 (n = 227)	160 (85.0)	47 (70.2)	14.8 (2.6 to 27.1)^*^	0.01^*^
Freestanding ED #2 (n = 181)	85 (73.9)	44 (66.7)	7.2 (−6.7 to 21.2)	0.30

* Indicates a statistically significant difference.

*CI*, confidence interval; *ED*, emergency department.

When redefining day shift by departure time, there were 492 day-shift patients and 508 night-shift patients. Of those, 396 (80.5%) and 357 (70.3%) were promoters for the day and night shift, respectively, a difference of 10.2% (95% CI 4.9–15.6%), *P* < 0.001.

When analyzing the data by eight-hour epochs, 307 (80.2%) of 383 patients who arrived between 6 am–2 pm were promoters. Meanwhile, 339 (75.5%) of 449 patients who arrived between 2 pm–10 pm were promoters, and 107 (63.7%) of 168 who arrived between 10 pm–6 am were promoters. Combining the eight-hour day and swing shifts together, 77.6% of surveys were promoters, which is 13.9% (95% CI 6.2–21.8%) higher than the eight-hour night shift group, *P* < 0.001.

Nineteen completed surveys came from patients seen by one of our three nocturnists. Of those, 12 (63.2%) were promoters. Additionally, the one physician who only worked day shifts had 24 completed surveys, of which 21 (87.5%) were promoters. Excluding the combined 43 encounters from that physician and the three nocturnists made it such that 78.8% of patients who arrived between 7 am–7 pm were promoters and 68.2% of the patients who arrived between 7 pm–7 am were promoters, a difference of 10.6% (95% CI 4.6–16.6%), *P* =  < 0.001.

### Multivariable Regression Analysis

On multivariable analysis, arrival during the night shift had a statistically significant association with a lower chance that the patient would be a promoter, with adjusted odds ratio 0.60 (95% CI 0.43–0.84), *P* = 0.003. Other than night-shift arrival, no other variables were associated with a reduced chance of being a promoter. On the other hand, elderly patients (age > 65) and non-English speaking patients had positive associations with being a promoter (Table 4).

**Table 4.** The adjusted odds ratios of various demographic and clinical variables and their association with being a “promoter” (a patient who gives high ratings to a physician on patient satisfaction surveys).

Characteristic	Adjusted odds ratio for being a promoter (95% CI)
Demographics	
Elderly (age > 65) (n = 281)	2.62 (1.72–4.08)^*^
Pediatric (age < 18) (n = 106)	0.80 (0.48–1.34)
White race (n = 778)	0.91 (0.62–1.31)
Hispanic ethnicity (n = 570)	0.86 (0.59–1.26)
Health Insurance	
Commercial (n = 594)	0.78 (0.53–1.14)
Government or other	Reference
No insurance (n = 101)	0.83 (0.47–1.46)
Married (n = 366)	0.98 (0.70–1.40)
Non-English speaking (n = 341)	1.82 (1.18–2.82)^*^
Time of year of visit	
Quarter 1 (n = 237)	0.93 (0.60–1.43)
Quarter 2 (n = 273)	0.84 (0.55–1.28)
Quarter 3 (n = 240)	1.29 (0.82–2.03)
Quarter 4 (n = 250)	Reference
Facility	
Main ED (n = 592)	Reference
Freestanding ED #1 (n = 227)	0.91 (0.38–1.08)
Freestanding ED #2 (n = 181)	0.65 (0.51–1.60)
Clinician characteristics	
Seen by a resident (n = 397)	0.81 (0.51–1.27)
APP primarily (n = 207)	0.78 (0.51–1.19)
Sign-out (n = 54)	1.08 (0.54–2.28)
Clinical characteristics	
Emergency severity index	
ESI level 2 or 3 (n = 663)	Reference
ESI level 4 or 5 (n = 337)	0.97 (0.67–1.41)
Arrived by ambulance (n = 46)	0.54 (0.28–1.09)
Blood test performed (n = 413)	1.11 (0.76–1.64)
Advanced Imaging performed (n = 318)	1.38 (0.92–2.09)
Opioid pain medicine given (n = 108)	1.01 (0.60–1.73)
Prescription given (n = 543)	1.15 (0.84–1.59)
ED length of stay (for 1-h increase)	0.94 (0.84–1.04)
Arrival during night shift (n = 337)	0.60 (0.43–0.84)*

* Indicates a statistically significant association.

*APP*, advanced practice practitioner; *CI*, confidence interval; *ED*, emergency department; *ESI*, Emergency Severity Index.

## DISCUSSION

In this cross-sectional study, we found that ED patients who arrive or depart during night shift are less likely to be promoters as compared to day-shift patients. Notably, the patient population that completed the satisfaction surveys and arrived during a night shift was substantially different than those patients who arrived during day shift. Considering this and other intuitive challenges of night shifts, unadjusted comparisons of physicians who work different ratios of day and night shifts on any number of metrics are likely to be compromised. However, in our study even after adjusting for several differences between day and night shifts, we still found an association between night-shift arrival and lower patient satisfaction scores.

Prior data on this subject has been mixed. One prior study evaluated the relationship between night shifts and ED patient satisfaction scores using data from 2009–2013 from a single ED and did not show a significant association.^14^ On the other hand, a prior large study using data from 42 facilities from 2012–2015 found that physicians who worked fewer night shifts had higher patient satisfaction scores.^16^ Both of these studies attempted to assess a large number of physician, facility, and operational factors that might affect patient satisfaction scores. A relative strength of our study was that it was a more targeted and granular assessment specifically of night- vs day-shift patient satisfaction scores.

Our study was not designed to specifically assess the associations of other variables with patient satisfaction scores, but we will briefly review the secondary findings. In this regard, our results were largely consistent with previous work, including our findings that elderly patients and non-English speaking patients are more likely to provide high satisfaction scores.^14^ ^,^ ^22^ Prior data has been mixed with regard to the association of LOS and patient satisfaction.^11^ ^–^ ^13^ ^,^ ^16^ ^,^ ^24^ ^,^ ^25^ We failed to find an association between ED LOS and patient satisfaction (Table 4), which is consistent with previous work that has reported that perceived LOS is more important than actual LOS.^24^ ^,^ ^25^

Overall, although empathy and communication are important contributors to patient satisfaction^15^ ^,^ ^22^ ^,^ ^24^ that an emergency clinician can mostly control, there are many factors that they cannot. Our data and previous demonstrate that night shift work, the patient population,^14^ and the facility^16^ ^,^ ^23^ all influence patient satisfaction scores. Considering also that the response rate for ED satisfaction surveys is so low (<10% in our system and similar in many others^26^) and that only discharged patients are sent surveys, we recommend against the use of patient satisfaction scores to determine payment for emergency clinicians.

## LIMITATIONS

This study had several limitations. First, our data comes from a single hospital system that has fairly high patient satisfaction scores; so, our results may not be applicable to other EDs. Additionally, given the retrospective and observational nature of the study, there could have been some unmeasured confounders that could explain the differences in patient satisfaction between the day and night shifts. Namely, while physicians usually work both day and night shifts, nursing staff and support staff more typically work only days or only nights. Therefore, differences in staffing might explain the differences in satisfaction scores. Moreover, prior studies have demonstrated that communication is an important factor in ED patient satisfaction scores,^22^ ^,^ ^27^ but given the design of this study, it was not possible to assess the quality of communication.

Next, our data did not have the granularity to adjust for patient volume for each shift, which could have impacted patient satisfaction. However, given that the median ED LOS was similar in the day- and night-shift groups, we doubt that differences in patient volume would explain the lower satisfaction scores by night-shift patients. Lastly, the low response rate to patient satisfaction surveys is a limitation in that survey responses are likely substantially influenced by selection bias, but we do not consider this a limitation specific to our study because our goal was to compare the real-world patient satisfaction scores from day- vs night-shift patients (with current survey techniques). Our results thus provide a comparison of the data that is actually being used to assess clinicians’ performance on patient satisfaction.

## CONCLUSION

In this cross-sectional study, night-shift arrival to the ED was associated with a statistically significant lower chance that the patient would be a promoter on satisfaction surveys. Given this finding and previous data suggesting that other issues beyond the physician’s control heavily influence satisfaction scores, facility factors, patient characteristics, and operational factors (including the time of the ED visit) should be considered when assessing patient satisfaction scores.

## Supplementary Information




