# Supplementary material for: Emergency Department Patient Satisfaction Scores Are Lower for Patients Who Arrive During the Night Shift
Source: West J Emerg Med. 2024 Oct 2;25(6):929–37. doi: 10.5811/westjem.20326 (PMC11610739; doi:10.5811/westjem.20326)
Supplement: Supplementary file 1 [file wjem-25-929-s001.docx]

**Patient Satisfaction Study Data Dictionary and Data Collection Instructions**

1. Search for the patient encounter in Epic using the coded MRN and visit date. Make sure you select the correct visit date (which for this study should always be in 2022). Fill in each of the empty columns using the information and definitions below.

2. For the **Arrival Instant** and **Departure Instant** columns, record the time the patient arrived to the ED and the time they departed from the ED as mm/dd/yyyy followed by time. For example, 01/05/2022 10:45. This information is shown if you hover your mouse over the word “Discharged” on the left side of the screen. The **Length of Stay** column automatically calculates the ED length-of-stay by subtracting the arrival instant from the departure instant.

3. For the **Ethnicity** column, write “Hispanic” or “non-Hispanic”. You can determine this by hovering over the patient’s name on the left side of the screen. Ethnicity will be listed directly below Race. If no ethnicity is recorded, write “NA”.

4. For the **Insurance** column, write “Commercial”, “Government only”, “None”, or “Other”. You can determine the patient’s insurance by looking in their facesheet. Any form of Medicare, Medicaid, or Children’s Health Insurance Program should be considered governmental insurance. If a patient has both commercial and governmental insurance, write “Commercial”. If the patient has no insurance, write “None”. If the patient has an insurance listed that does not seem to be commercial or governmental such as military (VA) or Workers’ Compensation, write “Other”.

5. For the **Arrival Method** column, write “Ambulance”, “Walk-in”, or “Other”. You can find this information by clicking on the “triage” tab where you should see the “Means of Arrival” under “Triage Summary”. If no arrival method is recorded, write “NA”.

6. For the **Seen by resident** column, write “Yes” if a resident (of any sort) participated in the care of this patient, and write “No” if no resident participated in the care of the patient. You can determine this by reviewing if a resident signed the ED Provider Note or an ED Note for this encounter. Their name will appear above the attending’s name. Additionally, review the attending’s documentation to see if there was any mention that a resident participated in the care of the patient.

7. For the **Sign out** column, write “Yes” if the patient had a sign out (or change of shift) during their time in the ED, and write “No” if not. Consider a patient to have been subjected to a sign-out if a physician (including a resident) or an APP finished their shift before the ED care of the patient was complete as indicated by documentation by the physician or APP from the subsequent shift.

8. For the **ESI** column, look on the left side of the screen next to the words “total time”. There should be a number in a colored circle. Record “1”, “2”, “3”, “4”, or “5”. If there is no ESI recorded, write “NA”.

9. For the next several columns, click on the “Workup” tab. For **Blood test**, **Radiology Ultrasound**, **POCUS**, **CT scan**, and **MRI**, determine if each of these types of diagnostic test were performed. Write “Yes” if they were performed in the ED and “No” if they were not. Scan the ED Provider Note to see if a POCUS was done that was not put into Epic. Any blood test including a point-of-care glucose counts as a blood test. For **Other imaging (specify)**, write the name of the other test performed such as “HIDA” or “VQ scan”. Consider an **Advanced Imaging** study to be a radiology ultrasound, POCUS, CT scan, MRI, or nuclear medicine scan. If they had any of these, consider the patient to have had an advanced imaging study.

10. For **Opioid** **Pain Medicine**, review the medications administered to the patient in the ED, and write “Yes” if they received any sort of opioid pain medicine. Opioid pain medicines include morphine, fentanyl, Dilaudid (hydromorphone), Percocet (oxycodone), Norco (hydrocodone), and Ultram (tramadol).

11. For **In-person evaluation by consultant**, write “Yes” if a consultant of any specialty evaluated the patient in the ED for this encounter. Write “No” if not. A primary care doctor or general internal medicine physician counts as a consultant. This information can be found by looking at the orders to see if a consultation order was placed as well as by looking to see if there is a note from a consultant documented for this encounter. As some consultants may have seen the patient without a consult order and may not have documented their evaluation, search the ED nursing notes and the ED Provider Note to see if there is any mention that an in-person consultant evaluation occurred.

12. For **phone consultation**, write “Yes” if a consultant of any specialty was contacted by phone call or text message about this patient during their ED encounter. Write “No” if not. A primary care doctor or general internal medicine physician counts as a consultant. This information can be found by searching the ED Provider Note to see if there is any mention that an ED provider spoke to a consultant during the encounter.

13. For **Given Rx**, click on the “Dispo” tab. Write “Yes” if they received any prescription, and write “No” if they received no prescription.

14. For **Disposition**, write “Discharged”, “AMA”, “Eloped”, “LBT”, or “Transferred”. This information is found in the “Dispo” tab. Patients who left before being seen by a physician or APP should be listed as LBT (left before treatment). Patients who are categorized as AMA, Eloped, or LBT will be grouped together for analysis.
